# Stray dogs as indicators of *Toxoplasma gondii *distributed in the environment: the first report across an urban-rural gradient in China

**DOI:** 10.1186/1756-3305-5-5

**Published:** 2012-01-05

**Authors:** Chao Yan, Lin-Lin Fu, Cai-Ling Yue, Ren-Xian Tang, Yi-Sheng Liu, Liang Lv, Na Shi, Ping Zeng, Peng Zhang, Dong-Hui Wang, Dong-Hui Zhou, Xing-Quan Zhu, Kui-Yang Zheng

**Affiliations:** 1 Department of Pathogen Biology and Immunology, Laboratory of Infection and Immunity, Xuzhou Medical College, Xuzhou, Jiangsu Province, China; 2 State Key Laboratory of Veterinary Etiological Biology, Key Laboratory of Veterinary Parasitology of Gansu Province, Lanzhou Veterinary Research Institute, Chinese Academy of Agricultural Sciences, Lanzhou, Gansu Province, China; 3 Grade 2008, the First Department of Clinical Medicine, Xuzhou Medical College, Xuzhou, Jiangsu Province, China; 4 Department of Epidemiology and Healthy Statistics, Xuzhou Medical College, Xuzhou, Jiangsu Province, China; 5 College of Animal Science and Technology, Yunnan Agricultural University, Kunming, Yunnan Province, China; 6 College of Animal Science and Veterinary Medicine, Heilongjiang Bayi Agricultural University, Daqing, Heilongjiang Province, China

**Keywords:** Prevalence, *Toxoplasma gondii*, Stray dog, Enzyme-linked immunosorbent assay (ELISA), Environment, Indicator

## Abstract

**Background:**

Toxoplasmosis is an important parasitic zoonosis caused by the protozoan *Toxoplasma gondii *that is distributed world-wide and infects a variety of hosts. However, the prevalence of *T. gondii *in the environment (such as soil, water and food) is largely unknown. Due to the technical difficulty in oocyst counting directly, an alternative assay using the serologic status of *T. gondii *in free-living animals, such as stray or free-living dogs, as an indicator, can be used to evaluate environmental contamination indirectly, as they are exposed to the same risk of infection as humans and other animals.

**Results:**

In the present study, 231 stray or free-living dogs across an urban-rural gradient were examined to assess the frequency of *T. gondii *in the environment. Specific antibodies to *T. gondii *were found in 93 dogs (40.3%) by enzyme-linked immunosorbent assay (ELISA), and no statistically significant differences were observed in seroprevalences of *T. gondii *between urban dogs (38.7%) and rural dogs (41%) (*p *> 0.05).

**Conclusions:**

A high seroprevalence of *T. gondii *in stray or free-living dogs in the present study indicates that there would be a wide distribution and a constant infection pressure of *T. gondii *across an urban-rural gradient, and the oocysts of *T. gondii *in the environment would be an important source of infection for humans and other animals both in urban and rural areas in China.

## Background

Toxoplasmosis is an important parasitic zoonosis caused by the protozoan *Toxoplasma gondii *that is distributed world-wide and infects a variety of hosts including humans, domestic animals and birds [1]. In humans, it was estimated that 30% of humans in the world and 7.88% of population in China were exposed to *T. gondii*, respectively [2-4]. *T. gondii *is mainly acquired by ingestion of under-cooked meat containing tissue cysts and water or food contaminated by oocysts in the environment [5,6]. Infection can also occur when *T. gondii *transmits from an infected mother to the fetus vertically [1,2]. However, it is still unknown which route of transmission is more prevalent for postnatal toxoplasmosis as no methods can differentiate oocyst-induced infection from those cysts formed, although progress is being made [7,8].

Felids, as the only known definitive hosts that shed environmentally-resistant oocysts into the environment, are essential in the life cycle and dispersal of *T. gondii *in the environment [9]. The shed oocyst is sporulated under favorable climatic conditions, and remains infectious for up to approximately 2 years, leading to a widespread environmental contamination and an important source for exposure of humans, domestic animals and wildlife to *T. gondii *[5,10-12]. In addition, the marine ecosystem can also be contaminated by environmentally resistant oocysts that are secreted by terrestrial felids and transported into the marine environment via freshwater runoff [13].

As oocyst of *T. gondii *in the environment is an alternative source for acquired toxoplasmosis and plays a significant role in the transmission dynamics of *T. gondii*, with constant efforts being made to seek a rapid, accurate and sensitive assay in determining the prevalence of *T. gondii *in the environment directly. Unfortunately, the progress is not satisfactory [14,15]. Thus, an alternative strategy using seroprevalence of *T. gondii *infecting free-living animals such as stray dogs which are considered as sentinels has been adopted for measuring the distribution of *T. gondii *exposure in environment indirectly [16-18].

Stray or free-living dogs are considered as the best indicators of *T. gondii *in the environment for the following reasons: I) stray dogs without any protection from pathogens roam freely and contact the same contaminated environment which humans are also exposed to; II) they are available both in urban and rural areas in practice, where the sanitary conditions may be very different; III) compared with other free-living animals, dogs have a keen sense of smell and have the behaviour of rolling in cats feces, increasing their exposure [19]; IV) canine toxoplasmosis can be also caused by ingestion of food meats containing cysts of *T. gondii*, but these food meats are mainly produced by non-carnivorous sources that are most likely to be infected by oocysts in the environment, such as birds, small mammals and under-cooked meat from humans' refuse. Interestingly, an epidemiological study showed a uniform prevalence between human toxoplasmosis and dogs exposed to *T. gondii *in the same region, indicating that stray dogs might be good sentinels of *T. gondii *exposure to humans and wildlife [2,17].

Climates of different geographical locations may also have a great influence on the transmission dynamics of *T. gondii *in the environment as they cannot become sporulated, survive and remain infectious without favorable climatic conditions [20,21]. However, little is known of the distribution and destiny of *T. gondii *in the environment of People's Republic of China (PRC) where the environmental hygiene of urban cities and rural areas are very different. Now, PRC is undergoing a rapid urbanization that may also affect the transmission and distribution of *T. gondii *oocysts in the environment [12]. In view of this background, the objectives of this study were to estimate the distribution and transmission dynamics of *T. gondii *in the environment by examining the seroprevalence of stray dogs as indicators, as well as identifying the risks of *T. gondii *in the environment that humans are exposed to in PRC.

## Results and Discussion

**Figure 1:**
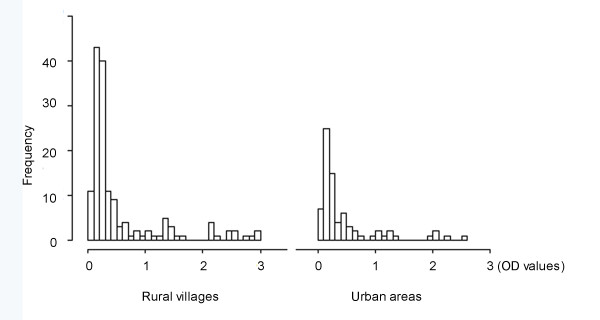
**Frequency distribution of individual optical density (OD) values by ELISA in different environmental conditions**.

**Figure 2:**
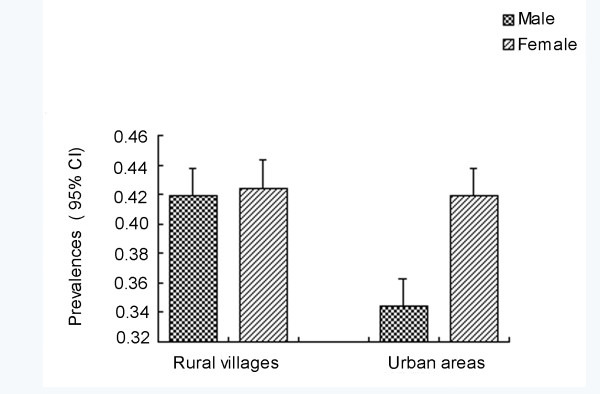
**Incidence rates of dogs exposure to *T. gondii *in different genders and geographical origins by ELISA**. Bars represent the 95% confidence interval of the measure.

**Figure 3:**
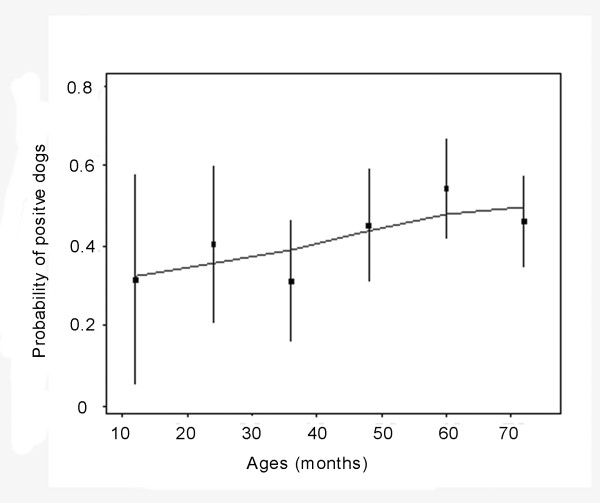
**Relationship between ages of dogs and the seropositivity of *T. gondii *exposure**. The animals were divided into 6 age groups (< 12, [12-60], ≥ 60 months) and lines represent estimated probability of seropositivity depending on age at 95% confidence intervals.

*T. gondii *antibodies were found in 93 (40.3%) of 231 dogs examined, and four dogs (1.7%) were considered "doubtful". The distribution of individual OD values are presented in Figure 1,and the antibodies levels of positive dogs were high, compared with OD values reported by Meireles *et al*. [17]. Table 1 shows that seroprevalence of *T. gondii *in rural village dogs is a little higher than those from urban areas, and the males were slightly lower than female ones, but no associations were found between origins, genders and the seropositivity of *T. gondii *in dogs (*p *≥ 0.05) (Table 1 Figure 2). However, the seropositivity of *T. gondii *increased with age, showing a relationship between the prevalence of *T. gondii *and ages of stray or free-living dogs, although this effect is weak (Figure 3).

**Table 1:** Prevalence of *Toxoplasma gondii *in dogs of different genders and geographical origins

Biometric data	Origins		Genders	
	
	Urban	Rural villages	Male	Female
Sample No.	75	156	121	110
Doubtful No.*	4	0	3	1
Positive No.	29	64	47	46
Prevalence (%)	38.7	41.0	38.9	41.8
Odds ratio [95% CI]	0.993 [0.51; 1.756]		1.051 [0.624; 1.770]	

* samples whose OD-value was between 0.9 times and 1.1 times of the mean OD value of critical controls according to manufacturer's recommendations.

The prevalence of *T. gondii *in the environment is largely unknown, and it is definitely associated with different geographical locations, climates, and animal welfare, which have a great influence on transmission of *T. gondii *[22,23]. In the present study, a high seroprevalence of *T. gondii *in stray or free-living dogs was obtained, and the seroprevalences in dogs of different origins did not present any statistically significant difference, indicating a wide distribution and a constant infection pressure of *T. gondii *in different environmental conditions. To illustrate this, a parallel study of 41 stray cats obtained 11 *T. gondii *isolates by bioassay in this region (unpublished data), therefore, ubiquitous stray cats infected by *T. gondii *are mainly responsible for the widespread distribution and constant infection pressure in the environment.

Four dogs with OD-value falling in between 0.9 times and 1.1 times of the mean OD-value of critical controls failed to be classified as positive or negative, and they were considered as "doubtful" for *T. gondii *infection according to manufacture's recommendations, suggesting that those animals were likely in acute phase or long chronic infection with *T. gondii *as the levels of antibodies to *T. gondii *were low and close to background [24,25].

In this study, the prevalence of dogs exposed to *T. gondii *increased with age, suggesting acquisition of infection rather than congenital transmission of *T. gondii *in the canine population, which is in accordance with reports by others [26-28]. It is interpreted that older dogs have more chance to feed on food or have contact with the surrounding environment that can be contaminated by *T. gondii *oocysts.

Attempts to evaluate the presence of *T. gondii *oocysts in the environment directly have been unsuccessful, although PCR assays are considered as the most promising methods for detecting *T. gondii *DNA in the environment directly [1,29-31]. Therefore, we chose stray or free-living dogs as sentinels of the distribution of *T. gondii *in the environment indirectly in the present study. Stray dogs of the present study were infected with *T. gondii *mainly via feeding on contaminated waters, small mammals and birds that are most likely infected by oocysts in the environment since they are non-carnivorous. Besides, ingestion of humans' leftovers including under-cooked animal meat that was produced on animal farms where toxoplasmosis of animals was probably neglected is another probable source for canine toxoplasmosis.

## Conclusions

A high seroprevalence of *T. gondii *in stray or free-living dogs in the present study indicates that there would be a wide distribution and a constant infection pressure of *T. gondii *across the urban-rural gradient, and the oocysts of *T. gondii *in the environment would be an important source of infection for humans and other animals both in urban and rural areas in China. Therefore, the knowledge of the distribution and transmission dynamics of *T. gondii *in the environment should be further recognized and explored as humans are likewise potentially at risk of exposure to *T. gondii*. Furthermore, measures that eliminate the sources of *T. gondii *in the environment, for example, the campaign of capturing stray dogs and cats and hygienic education programs for preventing *T. gondii *exposure to residents should be executed.

## Methods

### Ethics Statement

All dogs were handled in strict accordance with good animal practice according to the Animal Ethics Procedures and Guidelines of the People's Republic of China, and the study was approved by the Animal Ethics Committee of Xuzhou Medical College (No. SCXK<SU>2010-0003).

### The investigated regions

This study was performed in Xuzhou City which is located in Eastern China, and shares borders with Shandong Province, Anhui Province and Henan Province between 33°43'~34°58' North latitudes and 116°22'~118°40' East longitudes. The city covers an area of approximately 11,000 square kilometers and has a 9.4 million population. The climate of this region is a seasonal temperate semi-humid monsoon with an average annual temperature of 14°C and an average annual rainfall ranging from 800 mm to 930 mm. This information was available at http://www.xz.gov.cn/xzgl/zrdl/.

### Sampling of naturally infected dogs

The animals in the present study consisted of 75 stray dogs from a stray animal shelter in Xuzhou city, and 156 stray or free-living dogs slaughtered for animal meat, which originated from surrounding rural villages. Blood samples were collected randomly and the information such as sex, age and source were also obtained and matched. Blood was allowed to clot at room temperature, then centrifuged at 1800 *g *for 10 min. The sera were separated and stored at -20°C for further analysis.

### Serological assay

The presence of *T. gondii *antibodies was determined using a commercial ELISA kit according to the manufacturer's recommendations (Zhuhai S.E.Z. Haitai Pharmaceutlcals Co., Ltd, Zhuhai, China). This kit has been confirmed by specific PCR assay for detecting tissue cysts in experimentally infected pigs in our laboratory [32]. Briefly, sera diluted 1:100 were incubated in a *T. gondii *antigen-coated 96-well plate at 37°C for 30 min, and the plate was washed 3 times, then a drop of HRP-labelled conjugate was added into each well. After a final washing, "A" and "B" solution were then added and incubated at 37°C for 10 min. The reaction was blocked by adding a drop of "stop solution". The optical density (OD) at 450 nm was read using a photometer (BioTek Instrument Inc, Vermont, USA).

Positive control, negative control, three cut-off controls and blank control provided by the manufacturer were included in each test. All OD-values for the test sera were corrected according to blank controls, and OD-values of samples were authentic if the OD-value of positive control was > 0.6 and negative control was ≤ 0.2 in each test. The threshold value was determined by the mean of 3 critical controls in each test. Samples were considered positive if OD-values were greater than 1.1 times of threshold values and negative if OD-values were less than 0.9 times of threshold values, respectively. Those sera with the OD-values falling in between 0.9 times and 1.1 times of the threshold values were considered dubitable and should be re-tested.

### Statistical analysis

Data were analyzed by the chi-square test using Chi Square Test in SPSS for Windows (Release 16.0 standard version, SPSS Inc., Chicago, America) and excel 2003 (Microsoft^®^). Statistically significant difference was observed if the *p *value was < 0.05.

## Conflict of interest statement

The authors declare that they have no competing interests.

## Authors' contributions

Conceived and designed the experiments: XQZ, KYZ, CY and FFL. Performed the experiments: CY and FFL; Analyzed the data: PZ and CY; Contributed reagents/materials/analysis tools: CLY, RXT, YSL, LL, NS, PZe, PZh, DHW, DHZ. Wrote the paper: CY, QXZ and KYZ. All authors read approved the final version of the manuscript.
